# Conceptions of child development, assessment, and health among indigenous families in primary care: a qualitative study

**DOI:** 10.3389/fpubh.2026.1801093

**Published:** 2026-03-19

**Authors:** Camila Lucchini-Raies, Claudia Bustamante-Troncoso, Claudia Alcayaga-Rojas, Angélica Farías-Cancino, Dayann Martínez-Santana, Marcela González-Agüero, Solange Campos-Romero, Carolina Muñoz-Guzmán, Antonia Alarcón, Francisca Marquez-Doren

**Affiliations:** 1 School of Nursing, Faculty of Medicine, Pontificia Universidad Católica de Chile, Sigma Chapter Alpha Beta Omicron, Santiago, Chile; 2 School of Social Work, Faculty of Social Science, Pontificia Universidad Católica de Chile, Santiago, Chile; 3 School of Psychology, Pontificia Universidad Católica de Chile, Santiago, Chile

**Keywords:** child development, cultural competency, indigenous health services, primary health care, qualitative research

## Abstract

**Aim:**

This exploratory qualitative study examines Indigenous conceptions of child development, health, and developmental assessment from the perspectives of Mapuche, Aymara, and Diaguita families, together with primary health care professionals who provide care to Indigenous children in Chile.

**Methods:**

A qualitative study grounded in a constructivist paradigm was conducted using a rapid assessment approach. Semi-structured interviews were carried out with Indigenous families of children under nine years of age and with primary health care professionals working in urban and rural territories. Data were analyzed through thematic analysis, incorporating investigator triangulation and member checking to strengthen analytic rigor.

**Results:**

Indigenous families conceptualized child development as a relational, community-based, and holistic process, closely connected to territory, family networks, and cultural practices. Traditional medicine and home-based care were commonly used as first responses to childhood illness, while formal health services were sought primarily for more severe conditions. Both families and health professionals identified tensions with the biomedical model guiding primary health care, including culturally misaligned developmental assessment tools, limited intercultural competencies among professionals, and insufficient recognition of Indigenous identity within routine child health supervision.

**Conclusion:**

The findings reveal structural mismatches between standardized child health programs and Indigenous worldviews, with implications for equity, cultural safety, and the organization of primary health care. Addressing inequities in Indigenous child health requires care models that recognize cultural diversity, plural health knowledge systems, and community-based conceptions of child development.

## 1 Introduction

Health, illness, and well-being are socio-cultural processes whose meanings vary according to the historical, territorial, and political contexts of communities. While the dominant biomedical model conceptualizes health primarily as the absence of disease located within the biological body (1), other medical systems offer more holistic and situated understandings of well-being (2, 3). At a global level, Indigenous Peoples understand health as a state of harmony with their environment, encompassing not only biological dimensions but also family, community, spiritual, and territorial relationships (3, 4).

Childhood is likewise a socially and culturally situated concept. Nevertheless, there is international consensus in recognizing childhood as a critical life stage that underpins future well-being, learning, and social participation (5). Understanding child development therefore requires consideration of the systemic, political, and cultural factors that shape children’s life trajectories (6). Among Indigenous Peoples, child development and well-being are conceived holistically, integrating cultural, emotional, spiritual, and relational dimensions in close connection with family and community life (7–11). This period, however, is often marked by tensions for Indigenous families, who must reconcile the continuity and re-signification of their own cultural practices with the adoption of biomedical-oriented child-rearing guidelines that predominate in health systems across Latin America (6).

International organizations have documented persistent inequities affecting Indigenous children in the Americas, stemming from structural relationships of marginalization associated with ethnicity, gender, and social class (5, 12, 13). These inequities are not exclusive to the region. Globally, Indigenous populations experience poorer health conditions, reflected in higher infant mortality, greater prevalence of growth delay, and reduced access to essential services, as a result of historical and structural determinants that constrain development opportunities (12, 14). Evidence from countries such as Argentina and Australia indicates systematic gaps between Indigenous and non-Indigenous children in development, nutrition, mental health, and access to services, associated with persistent poverty, structural racism, and ongoing colonial policies (15, 16).

In Chile, 12.8% of the population self-identifies as belonging to an Indigenous people (17, 18), and 8.7% of children and adolescents are Indigenous (6, 19). Evidence shows that Indigenous children experience higher levels of poverty, reduced access to basic services, and poorer health indicators compared to non-Indigenous children, including greater nutritional risk and higher infant mortality (20–23). These gaps are exacerbated by discriminatory practices, the limited implementation of intercultural approaches, and the lack of disaggregated data to inform public policy (12, 19). Notably, much of this evidence is based on standardized biomedical indicators, which, while useful for population-level comparisons, present limitations for a situated understanding of child well-being. However, little is known about how these inequities are experienced in everyday child health supervision.

In response to this context, international organizations have emphasized the need to advance toward culturally appropriate, participatory, and respectful health systems that recognize Indigenous worldviews (12, 19). Child health supervision constitutes a key space of intercultural encounter, where diverse understandings of health, illness, development, and care converge between Indigenous families and health teams. The literature highlights that culturally meaningful assessments require sensitivity, dialogue, and respect, conceptualized through frameworks such as cultural safety (24), cultural humility (25), and critical cultural competence (26). Nevertheless, persistent gaps remain in the training of health professionals in these competencies (27).

Against this background, understanding how Indigenous families and primary health care teams conceptualize child development, health, and developmental assessment practices becomes essential.

The aim of this study was to explore and compare Indigenous families’ and primary health care professionals’ conceptions of child development, health, and developmental assessment within child health supervision in Chilean primary health care, across Mapuche, Aymara, and Diaguita contexts.

## 2 Materials and methods

### 2.1 Study design and data generation strategies

An exploratory qualitative study was conducted within a constructivist paradigm. The constructivist paradigm assumes that meanings of health, development, and care are socially and culturally constructed, making it particularly appropriate for examining Indigenous conceptions within intercultural health contexts. A rapid assessment methodology was employed, which has been widely used in public health to enable the timely collection and analysis of data and to generate contextually relevant situational diagnoses within limited timeframes (28). The rapid assessment approach is well suited to intercultural public health contexts, where timely, contextually grounded evidence is needed to inform practice and policy.

Data generation was organized into two complementary components. First, sociodemographic questionnaires were developed for families and health professionals, including age, sex, educational level, ethnic self-identification, and child health care trajectories. These instruments were reviewed and validated by representatives of the Chilean Ministry of Health to ensure cultural and technical appropriateness.

Second, in-depth semi-structured interviews were conducted. This technique was selected for its flexibility and capacity to explore conceptions, experiences, and meanings associated with Indigenous child development in specific socio-cultural contexts (29). Interview guides were informed by prior literature on Indigenous child development and intercultural health and were iteratively refined following early interviews to ensure contextual sensitivity and conceptual clarity. Separate interview guides for families and health professionals were developed based on the literature and validated by the Ministry of Health. Interviews were audio-recorded with participants’ authorization, respecting their preferred communication styles, timing, and settings, and were complemented with field notes documenting contextual elements, researcher impressions, and interaction dynamics relevant for interpretation.

### 2.2 Participants and inclusion criteria

#### 2.2.1 Families

Mothers, fathers, and primary caregivers of Indigenous children under nine years of age belonging to the Mapuche, Aymara, and Diaguita peoples were interviewed. Participants were users of primary health care services. At least eight interviews per Indigenous group were planned, with final numbers adjusted according to thematic saturation. Inclusion criteria included being over 18 years of age, caring for an Indigenous child under nine years old, speaking Spanish, and participating in child health supervision within primary care.

#### 2.2.2 Health professionals

Health professionals providing care to Indigenous children in primary health care settings were interviewed. Interviews were conducted in person or via the Zoom platform. A minimum of six interviews was initially planned, with additional interviews conducted until thematic saturation was reached. Inclusion criteria included providing clinical care to Indigenous children and having been invited through coordination with the Ministry of Health.

### 2.3 Study context and recruitment procedures

Participants were selected using purposive quota sampling to ensure representation across Indigenous peoples (Mapuche, Aymara, and Diaguita), territories (urban and rural), and professional roles within primary health care. Quotas were defined in advance to enable comparative analysis across cultural and territorial contexts. Final sample size was guided by thematic saturation within each group.

Recruitment was coordinated with the Ministry of Health due to limitations in obtaining authorization directly from local health facility directors. Institutional authorization was managed through formal communications and reported to the Ethics Committee through procedural amendments.

A dual-entry recruitment strategy was implemented to safeguard voluntariness and cultural appropriateness of participation. This approach aimed to prevent potential institutional pressure, respect community mediation processes, and ensure that participation was perceived as autonomous and culturally safe. One pathway was coordinated through the Ministry of Health, while a second pathway involved community referents familiar to participants.

For health professionals, initial contact was facilitated by the Ministry of Health and national programs such as Chile Crece Más. Subsequently, all participants completed the sociodemographic questionnaire and communicated their interest in participating directly to the research team, ensuring that neither the Ministry nor the Health Services were informed of individual participation decisions. Interviews were scheduled by the research team.

### 2.4 Researcher positionality and reflexivity

The research team is composed of interdisciplinary professionals from nursing and social sciences, affiliated with a Chilean university and collaborating with national Indigenous Health Programs. The study was conducted in partnership with Ministry of Health counterparts, and reflexive discussions were held throughout data analysis to examine how researchers’ disciplinary and socio-cultural positions might shape interpretation. This reflexive process sought to enhance transparency and analytic rigor in the study of intercultural health dynamics.

## 3 Results

### 3.1 Participant characteristics

Between October 2, 2023, and January 15, 2024, a total of 35 semi-structured interviews were conducted: 28 with Indigenous families and 7 with health professionals. In two family interviews, both parents participated, resulting in a total of 30 adult family participants. In addition, one interview with health professionals was conducted as a group interview involving three professionals. Overall, the study included 39 individual participants (30 family members and 9 health professionals). Although six interviews with health professionals were initially planned, data collection was extended until thematic saturation was achieved, resulting in additional interviews. The distribution of participants and number of interviews is presented in Table 1.

**Table 1:** Distribution of participants and number of interviews conducted.

Indigenous group	Target group	No. of interviews	No. of participants
Diaguita	Families	8	8
	Health professionals	3	3
Mapuche	Families	12	14
	Health professionals	1	3
Aymara	Families	8	8
	Health professionals	3	3
Subtotal	Families	28	30
	Health professionals	7	9
Total		35	39

The mean age of family participants was 35.6 years (range: 25–47). Most participants were mothers (*n* = 27). Fourteen family participants formally self-identified as belonging to an Indigenous community. The predominant family structure was biparental (*n* = 27). Families had between one and four children, comprising a total of 44 children under nine years of age across all participating households.

Regarding health professionals, seven semi-structured interviews were conducted, including one group interview involving three professionals. In total, nine health professionals participated, distributed equally across three regions: Araucanía (*n* = 3), Atacama (*n* = 3), and Arica and Parinacota (*n* = 3). Participants represented diverse professional backgrounds involved in Indigenous child health care, including professionals responsible for child health supervision in primary health care facilities and early childhood educators working in early stimulation rooms. This disciplinary diversity enabled the collection of perspectives from multiple levels of intervention.

The mean age of health professionals was 34.3 years (range: 25–62), with an average of 9.3 years of professional experience. Although six of the nine professionals self-identified as Indigenous, they described similar tensions when applying standardized biomedical assessment tools, highlighting the structural rather than individual nature of these constraints. The geographic distribution and professional profile of health professionals is presented in Table 2.

**Table 2:** Geographic distribution and professional profile of health professionals.

Region/Professional	Total No.
Arica y Parinacota region	3
Early childhood educator	1
Nurse	2
Atacama region	3
Nurse	2
Nutritionist	1
Araucanía region	3
Nurse	1
Physician	1
Nutritionist	1
Total	9

### 3.2 Description of findings

Analysis revealed Indigenous conceptions of children’s holistic development from the perspectives of families and health professionals involved in their care. Through the qualitative analytic process, four interrelated categories were identified: Child-rearing experiences and generational changes, Perspectives on growth and child development, Health practices and responses to illness, and Relationships with the health system. Together, these categories illuminate how meanings of child development are constructed, negotiated, and sometimes contested between family-based, cultural, and biomedical knowledge systems. These categories are presented below with illustrative quotations.

#### 3.2.1 Child-rearing experiences and generational changes

Families from all three Indigenous peoples described significant transformations in child-rearing practices associated with urbanization, migration, and increased access to technology. These transitions were perceived as altering children’s play environments, autonomy, and the role of the community as a socializing agent.

Among Aymara families, participants described traditional childhood as characterized by greater freedom, close contact with nature, and early participation in productive activities. Mothers emphasized that contemporary urban life has reduced communal spaces and prioritized formal education:

*“Being an Aymara child meant being freer… with a lot of contact with nature and hard work (…) With my children it’s not the same anymore… they live in a more ‘civilized’ way, and I have prioritized that they study.”* (FAM4)

Diaguita families, particularly women, highlighted changes in gendered expectations for girls. Whereas girls were previously expected to assume domestic and caregiving responsibilities at an early age, current practices emphasize autonomy and self-development:

*“When I was a child, I had to learn how to be a mother with my nephews (…) no one had to tell me I had to learn; I learned it as a child.”* (FDM1)

Mapuche families valued continuity in community-based life, while acknowledging that technology has altered family dynamics and daily routines. Participants emphasized the importance of outdoor play and the sense of safety provided by rural communities:

*“Here there is more freedom (…) my children go out, get dirty, get muddy, but they are free and not in danger.”* (FMM1)

These transformations were described in ambivalent terms. While some families perceived urbanization and formal education as opportunities for social mobility and security, others framed them as processes associated with weakened community bonds and reduced intergenerational cultural transmission. Rather than representing simple cultural loss, these generational shifts were articulated as complex processes of negotiation, in which families actively reconfigure Indigenous child-rearing practices within shifting urban, educational, and technological contexts.

#### 3.2.2 Perspectives on growth and child development

Across all groups, Indigenous families emphasized community-based child-rearing as central to children’s well-being. In Diaguita families, the extended family was described as a key transmitter of values such as respect, patience, and tolerance, which guide everyday learning and coexistence:

*“I wasn’t disrespectful with my mother (…) there always has to be respect.”* (FDM1)

Families also highlighted children’s freedom to play in open spaces, contrasting rural environments with urban settings perceived as restrictive:

*“Down (in the city) there everything is confinement (…) here they are free to go out and play (…) he is happy.”* (FDM2)

Within the Mapuche worldview, children were described as complete persons who grow simultaneously in physical, mental, and spiritual dimensions. Their development was expressed through progressive participation in household and agricultural activities as part of community life:

*“Children who work in the countryside know how to do many things (…) it’s another way of raising children, not better or worse, just reality.”* (FMM4)

Aymara families, particularly in urban contexts, identified migration as a key factor disrupting intergenerational cultural transmission and weakening community bonds:

*“People migrate a lot from my community (…) those ties are lost (…) sometimes you don’t have that bond or attachment.”* (FAM7)

In contrast to the biomedical approach, which conceptualizes child development through standardized milestones, Indigenous families understood development as a dynamic process shaped by communal learning and cultural engagement. Health professionals reported difficulties applying psychomotor development assessment tools due to their limited cultural relevance:

*“The instruments are outdated (…) children here don’t recognize an alarm clock (…) I had to create my own materials so they could respond.”* (PAE2)

Across the three Indigenous groups, child development emerged as a relational and community-embedded process, grounded in collective life and territorial belonging. This understanding contrasts with the individualistic and milestone-based assumptions that underpin standardized biomedical assessment frameworks.

#### 3.2.3 Health practices and responses to illness

Care practices during illness reflected the coexistence of traditional knowledge systems and biomedical care. Aymara mothers described initially using medicinal herbs grown in household gardens and knowledge transmitted through family networks for mild symptoms, seeking formal health services only when conditions worsened:

*“Herbs work for me… if it becomes very serious, then I go to the doctor.”* (FAM1)

Diaguita mothers similarly prioritized home remedies understood as traditional therapeutic practices before administering pharmaceutical treatments:

*“First I use the remedies my mother and grandmother taught me (…) if that doesn’t work, then I go to medicine.”* (FDM6)

Among Mapuche families, the use of *lawen* (traditional medicine) was embedded within spiritual and emotional understandings of illness:

*“Until the Machi told me to make an infusion with matico and bathe him with oats…”* (FMM8)

Food practices also emerged as a point of divergence between traditional practices and health system recommendations. Some families maintained culturally rooted diets that did not always align with nutritional guidelines provided by health centers:

*“Health professionals have adapted to recommending foods we actually have here… the diet that guides us as Aymara people… which isn’t the same as what they recommend at the health center.”* (FAM1)

Health professionals acknowledged the importance of providing education without imposing biomedical norms, emphasizing respectful dialogue:

*“We try to avoid imposing things (…) the idea is to educate.”* (PDE1)

These accounts illustrate a context of therapeutic pluralism in which traditional and biomedical systems coexist. Rather than rejecting formal health services, families described strategically navigating multiple knowledge systems according to the perceived severity and meaning of illness.

#### 3.2.4 Relationships with the health system

Experiences with the health system varied by territory, type of facility, and degree of intercultural integration. Aymara families valued the care received but described it as routine and insufficiently responsive to their cultural identity:

*“The care is routine… I would have liked them to strengthen Aymara culture more.”* (FAM1)

Diaguita families reported difficulties accessing traditional healers within hospital settings:

*“I needed them to call the Meica because she would assist my birth (…) but they didn’t do anything.”* (FDM2)

Mapuche families attending intercultural health centers described more positive experiences, characterized by attentive listening and adequate consultation time:

*“The Filulawen Intercultural Health Center has always provided different care (…) they take the time to listen.”* (FMM4)

By contrast, urban health centers were perceived as offering more standardized and culturally misaligned care. Health professionals acknowledged gaps in their intercultural training and limited knowledge of Indigenous dietary practices, beliefs, and family priorities:

*“There should be more training (…) sometimes we try to impose things they don’t actually do.”* (PDE1)

Professionals also highlighted the lack of systematic recording of Indigenous identity in clinical records, limiting continuity of intercultural care:

*“It’s recorded at registration… but during check-ups, no professional asks about it.”* (PDE2)

Experiences with the health system were shaped by the degree of intercultural integration across territories and facilities. Attentive listening, recognition of Indigenous identity, and flexibility in care practices emerged as central elements shaping trust and perceived quality of care.

## 4 Discussion

This study provides a qualitative, comparative analysis of Indigenous families’ and health professionals’ conceptions of child development within primary health care across Mapuche, Aymara, and Diaguita contexts. Four interrelated findings emerged: (1) generational transformations in child-rearing understood as processes of cultural negotiation; (2) relational and territorially grounded conceptions of child development; (3) therapeutic pluralism in health practices; and (4) differentiated experiences with the health system shaped by the degree of intercultural integration.

Consistent with previous research, our findings show that Indigenous child-rearing practices and understandings of development are embedded in collective, cultural, and territorial relationships rather than in isolated individual milestones (8–11). However, by comparatively integrating three Indigenous peoples and incorporating both family and professional perspectives, this study further demonstrates how these relational frameworks frequently contrast with the standardized and individualizing logic that structures biomedical child health supervision in primary care.

Generational transformations in child-rearing practices, particularly those associated with urbanization, formal schooling, and technological change, were described as processes of adaptation and negotiation rather than simple cultural erosion. Families articulated both opportunities and tensions within these shifts, reflecting ongoing efforts to sustain cultural continuity while navigating dominant social and institutional expectations. This interpretation aligns with evidence indicating that modernization and migration reshape Indigenous parenting practices, often reconfiguring family and community roles while generating dynamic tensions between cultural continuity and adaptation (10, 30).

Beyond generational change, the relational foundations of child development emerged as a central organizing principle across Indigenous groups. Among Diaguita and Mapuche families, child-rearing was consistently described as a collective process in which the extended family plays a central role in transmitting values, norms, and everyday learning. This understanding aligns with studies that emphasize the importance of community life, intergenerational respect, and children’s progressive participation in domestic and productive activities as integral components of holistic child development (31, 32). In particular, the Mapuche conception of children as complete persons who grow simultaneously in physical, mental, and spiritual dimensions reflects a holistic worldview that challenges the fragmented and standardized approaches characteristic of the biomedical model.

For Aymara families, especially those residing in urban contexts, migration emerged as a central factor contributing to the weakening of community ties and disruptions in intergenerational cultural transmission. Previous research has similarly documented that migration processes negatively affect Indigenous families’ sense of belonging, attachment, and continuity of cultural practices related to early childhood (30). International studies further indicate that Indigenous mothers identify early child-rearing as a strategic space for sustaining cultural identity in highly institutionalized urban environments (33, 34).

Regarding health practices and responses to illness, the findings reveal the coexistence of traditional Indigenous medicine and biomedical care. Families across the three Indigenous groups reported initially using medicinal herbs, home remedies, and traditional healing practices, turning to formal health services mainly when illnesses were perceived as severe. This form of therapeutic pluralism has been widely described in Indigenous health literature, where traditional practices are recognized as legitimate and meaningful components of everyday care rather than as barriers to accessing biomedical services (35–37). Systematic reviews suggest that interventions aimed at improving Indigenous child health are more effective when they integrate cultural knowledge, strengthen extended family roles, and adopt community-centered approaches (38).

A particularly relevant finding is the shared perception among families and health professionals regarding the limited cultural relevance of standardized child development assessment tools used in primary health care. These instruments often rely on implicit cultural assumptions related to play, language, and everyday objects, which may lead to Indigenous children being classified as “at risk” or “delayed” in ways that do not reflect their lived contexts. Similar concerns have been raised in studies questioning the cultural validity of psychomotor development assessment tools in Indigenous and culturally diverse populations (10, 31). This misalignment generates tensions for health professionals, who are required to apply tools they perceive as culturally decontextualized. Notably, although several interviewed professionals self-identified as Indigenous, they reported similar constraints when applying standardized assessment instruments. This suggests that tensions are embedded within institutional structures rather than solely reflecting individual practitioners’ cultural positioning.

In relation to experiences within the health system, families described substantial differences depending on the type of facility. Intercultural health centers were valued for offering more respectful and responsive care, characterized by greater listening, longer consultation times, and explicit recognition of Indigenous worldviews. In contrast, urban primary health care centers were perceived as providing more standardized and culturally insensitive care. These findings are consistent with research conducted in Chilean primary care settings, which highlights gaps between the formal recognition of intercultural health policies and their effective implementation in everyday clinical practice (19).

From the perspective of health professionals, the results highlight the need for stronger training in intercultural health and improvements in clinical information systems, particularly regarding the systematic recording of Indigenous identity. The absence of this information limits the ability to plan culturally appropriate care and reinforces standardized biomedical approaches. International evidence supports these findings, indicating that insufficient cultural competencies and the invisibilization of Indigenous identity constitute structural barriers to equitable access and continuity of care for Indigenous children (18, 24, 38).

A practical implication that can be implemented immediately within primary health care settings is strengthening attentive listening during consultations. Families consistently associated feeling heard and respected with culturally safe care, suggesting that relational practices are not secondary but central to quality improvement.

From an implementation perspective, the findings suggest that improving Indigenous child health supervision requires structural changes that go beyond superficial adaptations of protocols or materials. In the short term, strengthening intercultural training for primary health care teams is essential. In the medium and long term, the co-design of child development assessment tools with Indigenous communities, the revision of national child health guidelines to incorporate culturally relevant indicators of well-being, and the strengthening of partnerships between primary health care teams and Indigenous organizations are critical steps. The contribution of this study lies in its comparative focus on three Indigenous peoples—Aymara, Diaguita, and Mapuche—and in integrating the perspectives of families and health professionals, offering evidence to inform more equitable, culturally safe, and contextually grounded primary health care models.

Taken together, these findings have direct implications for primary health care practice and policy. They highlight the need to move beyond standardized, deficit-oriented approaches to child health supervision and toward care models that are responsive to Indigenous ways of understanding child development, well-being, and caregiving. From a practice perspective, this involves strengthening intercultural competencies among health professionals, fostering respectful dialogue with families, and adapting assessment processes to reflect children’s social, cultural, and territorial contexts. From a policy perspective, the findings underscore the importance of revising child health guidelines to incorporate culturally relevant indicators of well-being, improving information systems to ensure the systematic recognition of Indigenous identity, and promoting the co-design of assessment tools and care strategies in partnership with Indigenous communities.

## 5 Conclusions and public health implications

This study demonstrates that Indigenous conceptions of child-rearing, child development, and child health among Mapuche, Aymara, and Diaguita peoples are grounded in relational, community-based, and territorial understandings of well-being. These conceptions frequently come into tension with standardized biomedical approaches that guide child health supervision within primary health care. The experiences reported by families and health professionals reveal structural mismatches between current child health programs and Indigenous socio-cultural realities, particularly in the use of development assessment tools with limited cultural relevance, the insufficient integration of traditional knowledge systems, and the inadequate recognition of Indigenous identity within routine care processes.

The relational, territorial, and spiritual dimensions described by families constitute epistemological foundations of child well-being, rather than contextual variables to be superficially accommodated within biomedical frameworks. Recognizing these dimensions as foundational challenges dominant assumptions embedded in standardized child health programs and calls for deeper structural transformation.

The lack of cultural relevance in child health supervision not only marginalizes Indigenous knowledge but also contributes directly to the reproduction of health inequities. When health services fail to align with families’ values, practices, and expectations, participation may decrease and opportunities for early and meaningful support may be lost. These findings underscore the importance of recognizing Indigenous child-rearing practices as legitimate, protective, and central to child well-being, rather than framing them through deficit-based or risk-oriented perspectives.

From a public health perspective, the results highlight the urgent need to advance toward primary health care policies and care models that recognize cultural diversity not merely as a normative requirement, but as a core dimension of care quality, child development, and health equity. This entails critically examining the cultural assumptions embedded in early childhood development programs, promoting culturally appropriate assessment practices, and strengthening culturally safe care environments that foster trust, community participation, and continuity of care.

Key public health implications include strengthening intercultural training within primary health care teams, improving systems for the systematic recognition of Indigenous identity, and advancing collaborative approaches that integrate biomedical and traditional health systems. In the medium and long term, the co-design of child development assessment tools and the incorporation of culturally grounded indicators of well-being into national guidelines are essential steps toward reducing inequities. Collectively, these actions are necessary to advance more equitable, culturally safe, and contextually responsive primary health care systems.

### 5.1 Limitations

Although this study included families and health professionals from three Indigenous peoples and diverse urban and rural territories, the findings reflect specific contexts and do not capture the full heterogeneity within each Indigenous group or among other Indigenous peoples not included in the study. Participation was predominantly represented by mothers, with limited involvement of fathers and male caregivers. This gender imbalance may restrict the diversity of caregiving perspectives captured and highlights the need for future research exploring Indigenous fatherhood and masculinities in child-rearing practices.

Additionally, interviews were conducted in Spanish, which may have constrained the expression of deeper cultural meanings for participants whose first language is an Indigenous language. Recruitment through the health system may have favored the inclusion of families with stronger connections to primary health care services, potentially excluding those with more limited access or engagement. Finally, while the rapid assessment methodology facilitated the timely generation of contextually relevant evidence for public health decision-making, it may have limited the depth of interpretive analysis. Nevertheless, this approach was coherent with the study’s objectives and allowed for the production of actionable insights to inform intercultural health policy and practice.

## Data Availability

The raw data supporting the conclusions of this article will be made available by the authors, without undue reservation.
